# Exploring Healthcare Experiences of Transgender Individuals

**DOI:** 10.1089/trgh.2016.0021

**Published:** 2016-11-01

**Authors:** Katie A.E. Ross, Madelyn P. Law, Amanda Bell

**Affiliations:** ^1^ Department of Health Sciences, Brock University, St. Catharines, Ontario, Canada.; ^2^ Department of Family Medicine, McMaster University, Hamilton, Ontario, Canada.

**Keywords:** access to care, qualitative methods, transgender

## Abstract

**Purpose:** It has been widely noted that existing healthcare systems do not always function effectively for the transgender population. Despite existing healthcare barriers, however, transgender individuals have been shown to have positive healthcare experiences. This study explored a cohort of transgender individuals who had positive healthcare experiences, and those who were involved in creating a positive healthcare experience for transgender individuals.

**Methods:** A single case study was conducted, which included 10 interviews with transgender individuals, healthcare providers, and friends/family/significant others of transgender individuals. Data were analyzed through thematic analysis.

**Results:** Seven key themes emerged within macro levels (large-scale system), meso levels (local/interpersonal), and micro levels (individual/internal) of healthcare system support. At a macro level, few system strengths were shown, with hope for change in the future. On a meso level, both external supports and informal networking emerged as key factors in positive healthcare experiences. At the micro level, self-navigation, characteristics for success, and personal strategy development were important for achieving positive experiences.

**Conclusion:** Factors that contribute to positive healthcare experiences for transgender individuals were outlined in this study, showing that meso and micro level support compensate for large-scale healthcare system deficits.

## Introduction

Transgender is an umbrella term that refers to people with diverse gender identities and expressions that differ from stereotypical gender norms.^1^ It has been widely noted that existing healthcare systems do not always function effectively for the transgender population.^2–5^

In Canada, transgender individuals face a number of health inequities that may impact their overall well-being. In an Ontario-wide study, more than half of transgender individuals reported symptoms consistent with clinical depression,^6^ and a further study by Bauer, Pyne, Francino, and Hammond showed that suicidal thoughts were seen in 36% of transgender individuals in Ontario, with 10% of participants attempting suicide within the past year.^7^ Transgender individuals also show an increased risk of acquiring the human immunodeficiency virus (HIV), with participants in an Ontario study self-reporting with HIV was shown to be 10 times greater than the baseline prevalence in the province.^8^

Trans PULSE, an Ontario-wide research study of social determinants of health among transgender individuals, has shown reports of stigma and discrimination,^9^ which can be seen as unacceptable in Canada, where the transgender population is covered under the Human Rights Act. Within the workplace, 13% of transgender individuals reported having been fired from their job for being transgender and 18% reported having been turned down due to a lack of transgender-positive attitudes and policies in the workplace.^10^ Transgender individuals in the Trans PULSE study also represent an underemployed population, with a median income of $15,000 per year, despite 44% of participants having an undergraduate or postgraduate degree.^8^

Within the healthcare setting, transgender individuals face a lack of sensitivity and respect, with 40% of transgender individuals in the Trans PULSE study reporting that they had faced discrimination (such as refusal of care, demeaning language, and refusal to examine specific body parts) from their family doctor.^11^ This results in transgender individuals avoiding encounters with the healthcare system; a finding that is reflected in the work of Bauer, Scheim, Deutsch, and Massarella, as they found that 21% of transgender individuals reported avoiding the emergency department when they needed it due to their transgender identity.^12^

On a system level, there has been a lack of standardization in the policies and services available to transgender individuals in Canada who make the decision to transition their bodies to more accurately represent their gender identity. Until 2002, Vancouver General Hospital's gender clinic was considered the sole provider of public health coverage for transition-related surgeries.^13^ With government funding cuts to this program, Vancouver Costal Health now provides system navigation support to transgender individuals, but no longer provides primary healthcare or surgical or hormonal readiness assessments to transgender people.^14^

Other notable organizations in Canada having a reputation for providing transgender care are Sherbourne Health Center in Toronto, which has developed guidelines and protocols for primary healthcare for transgender clients,^15^ and the Gender Reassignment Surgery Clinic in Montreal, which has a long-standing history of providing care to transgender individuals.^13^ These clinics are an excellent example of high-quality services geared toward this population; however, these specialized services are not always available to individuals in midsize urban and rural areas.

Coverage for gender-affirming surgical care varies from province-to-province with regard to which surgeries are covered and the amount of coverage provided.^13^ In Ontario, full coverage for transgender surgeries (including external genital surgery, hysterectomy, mastectomy, and augmentation mammoplasty)^16^ is now provided, after a 10-year delisting period since 2008.^17^ Funding for surgical procedures only covers the direct surgical costs, which accounts for roughly 25% of the overall cost of procedures.^13^ No coverage currently exists for surgery in New Brunswick, Nova Scotia, or Prince Edward Island.

In addition to funding issues surrounding surgeries, there is also an assessment process that transgender individuals must go through to be eligible for hormone therapy and/or surgery in Canada. Until March 2016, under suggestion of the World Professional Association for Transgender Health (WPATH) standards of care, Center for Addiction and Mental Health (CAMH) assessed transgender individuals seeking hormones and/or surgery.

Recent changes have expanded access for transgender individuals, allowing primary healthcare physicians and nurse practitioners to assess and refer individuals for transition-related funding.^18^ To qualify for coverage, transgender individuals must follow a set of criteria which, among other requirements, includes a diagnosis of Gender Dysphoria. Depending on the treatment sought (e.g., external genital surgery), individuals also require a “Continuous Gender Role Experience,” through which an individual must live in a role congruent with his or her identity and document this experience for 12 continuous months.^16^ The Continuous Gender Role Experience is no longer included in WPATH standards of care.^19^

## Satisfaction with Healthcare

Although it has been shown that there are many flaws in healthcare for the transgender population, it is imperative to note that there have been positive reports of patient satisfaction with healthcare among this population. In a needs assessment of transgender people living in Washington, DC, high levels of satisfaction with access to transgender-related care on a lifetime basis were found; however, it was also reported that overall access to care was low.^20^ Similar to these findings, Bockting, Robinson, Benner, and Scheltema found that despite access to care being an issue, once engaged in care, transgender individuals experienced high levels of satisfaction with their care at a university-based sexual health clinic.^21^

In transgender youth, satisfaction with services in general has also been noted, with 39% of respondents reporting that they had not received any unhelpful or negative services.^22^ A study looking to patient satisfaction with gender identity clinics in the United Kingdom found that despite embedded system barriers, transgender satisfaction with healthcare services can be achieved through clinical care that incorporates supervision of one's time living in their chosen gender role, provides adequate information about hormone treatment and postoperative hormone advice, provides support for significant others, friendly and courteous administration, and punctuality of both physicians and the scheduling follow-up appointments.^23^ Although these healthcare systems can be seen to differ from that of Canada, findings from these studies may be considered highly relevant, as literature has noted similar healthcare barriers for the transgender population across such developed countries.

Within Canada, transgender individuals have reported high levels of satisfaction with mental health services, with 87.8% of transgender-identified participants in a study comparing mental health services among the lesbian, gay, bisexual, and transgender (LGBT) community reporting that they were “satisfied” or “very satisfied” with their mental health services in the past year.^24^ These findings indicate that despite the many barriers that exist for the transgender population, there are in fact transgender individuals who have had positive healthcare experiences, warranting further research in this area.

## The Strengths-Based Approach

Taking the volume of deficits-based literature that exists in transgender healthcare into consideration, there is a clear need for further exploration of patient satisfaction with care in the transgender population, and what factors contribute to this satisfaction with care. The strengths-based approach provides a plausible lens through which to investigate this area, adopting the belief that there are many strengths and resources that an individual, group, or community possesses, which need to be fostered to benefit individuals, and society as a whole.^25^

Although there has been limited research looking exclusively to the transgender population with a distinct use of a strengths-based lens, one notable study found high levels of self-efficacy and many individual competencies when exploring life satisfaction among trans men in Vancouver, BC.^13^ Building on this concept, a strengths-based approach is used in the current study to explore a cohort of transgender individuals who had positive healthcare experiences, and those who were involved in creating positive healthcare experiences for transgender individuals.

## Methods

To explore positive transgender healthcare experiences, a qualitative case study was used. Well suited to this study, case study methodology allowed the highly important perceptions of transgender individuals to be uncovered and provided an understanding of the context in which these experiences occurred.

To recruit participants purposeful sampling was used, through which transgender individuals who had positive healthcare experiences, and those who contributed to these positive experiences, were considered information-rich and gave insight into what factors were important to positive healthcare experiences for this population. Through a snowball sampling approach, one initial transgender participant was recruited, who then referred other transgender individuals who might have had positive healthcare experiences, and those who had supported transgender individuals to have positive experiences (such as friends, family, significant others, and healthcare providers) to the study. The snowball then expanded, allowing for transgender participants to refer individuals they felt were relevant and could provide insight for the study. Before participant recruitment and data collection, Research Ethics Board approval was received through Brock University. To obtain trustworthiness in the study, credibility, transferability, dependability, and confirmability were established through adherence to Shenton's (2004) strategies for ensuring trustworthiness.^26^

### Data collection

Semistructured in-depth interviews were used to acquire information pertaining to the case. Interviews were conducted between February and May 2014, and ranged from 45 min to 2 h in length. An interview guide was used, with prompts for addressing key areas such as seeking and accessing health information and services, defining and providing examples of positive healthcare experiences, and supports for navigating the healthcare system. The interview guide was approved through a panel, which included experts in the areas of healthcare quality and human sexuality, as well as a family medicine doctor. The researcher conducting the interviews had an in-depth discussion with a transgender individual in the community regarding the suitability of the study and key content areas before data collection. All participant interviews were recorded and transcribed.

### Data analysis

Thematic analysis captured important patterns in the data. NVivo software was used to code and sort all data, allowing for the development of themes, and for researchers to assess the saturation of the data. A rich description of the data was incorporated, through which themes that were identified and analyzed represented the content of the entire data set, and gave an understanding of predominant themes. Transgender participants, healthcare providers, and friends/family/significant other participant groups were first analyzed separately to allow for relevant themes to emerge. Themes that surfaced then began to converge across participant groups, illuminating the case. To effectively conduct a strong thematic analysis, the phases of thematic analysis developed by Braun and Clarke were followed.^27^

## Findings

Findings in the current study describe the study population, define the positive healthcare experience for transgender individuals, and depict the levels through which transgender individuals are able to achieve positive healthcare experiences.

Participants in this research encompassed five transgender individuals (including four self-identified male-to-female individuals, and one female-to-male individual), three friends/family/significant others, and two healthcare providers (including a social worker and a psychotherapist). All transgender participants were Caucasian and between 18 and 65 years of age. In all participant groups, it was acknowledged that social factors directly influence the experience transgender individuals will have in the healthcare system.

In addition to the social stigma associated with identifying as transgender, which was seen as a reoccurring pattern expressed in the current study, participants largely discussed privilege, emphasizing that those transgender individuals with a number of layers of privilege (i.e., racial, employment, and economic privilege) were more likely to have positive healthcare experiences. While participants appeared to come from varying levels of privilege (all transgender participants were white, with varying income levels, employment status, and levels of education), all transgender participants in this study reported some positive healthcare experiences. Combined with previous research, this suggests that privilege can impact the experience of care but is not the sole determinant of positive healthcare experiences.

### Defining the positive experience

When asked to define a positive healthcare experience, provider characteristics were most commonly reported by transgender individuals. A provider's knowledge of transgender issues and relevant healthcare services was shown as a key element to positive experiences in the healthcare setting, as well as a provider's respect throughout the healthcare encounter. Stressing the importance of provider respect in the healthcare setting, one transgender participant stated:

I've had some experiences where the positive nature of the experience was simply the respect that I received from the people at the, you know the provider.

A provider's willingness to make referrals and the ability to have a connection with their transgender client were also seen as important factors in creating positive healthcare experiences. One transgender participant stated,

Well, the medical professional should be able to identify with you, compassion, willing to listen, avoiding the buzz words because you want to establish rapport, so I guess going back to the original point was having that connection. When you have that you have everything.

Participants in the study recognized that there were a number of supports that were important in order for transgender individuals to have positive healthcare experiences. These supports were described on a large-scale healthcare system level, within a local context, as well as on a personal level. This led to the delineation of three levels of support in the current study as macro, meso, and micro level healthcare support systems, with strengths that interconnect to encompass positive healthcare experiences for transgender individuals. Strengths seen on each of these system support levels are shown in Table 1.

**Table 1.** **Strengths at Macro, Meso, and Micro System Levels That Comes Together in Creating Positive Transgender Healthcare Experiences**

Macro		Meso		Micro	
Strength	Example	Strength	Example	Strength	Example
Policy	Rainbow Health Ontario seen to have positive impact on transgender healthcare policy	External support	Friends—share experiences, information, and advice, provide mentorship	Strategy development	Educate providers so that they will in-turn provide care
			Support groups—build social networks, serve as platform for sharing information and resources		Careful planning of one's healthcare journey
			Healthcare providers—provide positive experience through listening, normalizing the transgender experience, ensuring support systems in place, helping in informed decision making, writing letters of support, letting transgender individuals take change of their transition, self-educating		Viewing healthcare as a do-it-yourself project
Formal structure	Sick Kids new Toronto Gender Identity Clinic seen to be a positive change	Informal networking	Perceived to be taking place between individuals, providers, and organizations	Personality traits	Independence, patience, and persistence aid in system navigation
			Allow transgender individuals and friends to learn and share knowledge		

### The macro level

Although participants were asked to discuss the positive aspects in this level of support, it was evident that there were relatively few as they related to the system. It was important to acknowledge the barriers within the system, because they provided a framework for understanding how system deficits are overcome in meso and micro level support systems. Many deficits were seen with respect to healthcare policy, existing transgender healthcare structures, as well as formal provider training.

CAMH was noted as a main policy structure, with barriers to care embedded in the existing gender-affirming surgical care approval process. Participants emphasized that the approval process for both hormone therapy and gender-affirming surgical care was lengthy in terms of wait-time, and that the Gender Role Experience required by CAMH was frustrating. Finding appropriate ways to document this Gender Role Experience, as required by CAMH, also caused frustration because those individuals not enrolled in school or in the workforce expressed uncertainty around how to document their experience. A transgender participant highlighted the policy barriers that exist at CAMH by stating:

There are enormous barriers all over the place. I once saw one panel comic in the paper which was the sign at the wicket said DMV which is you know in the U.S. it's the department of motor vehicles, and the person is standing in the line and there's like a hoop of fire, and there's lions there and you know various other things, and the person at the wicket is saying “because we can,” you know, do you want your license or not? And so I took that and scanned it in and changed the wording to say “CAMH” and you know. That's what it felt like. It felt like they just put all these barriers in your way because they can.

Participants saw formal transgender healthcare organizations and infrastructure as very important for transgender individuals to have positive healthcare experiences. Such structures, however, were noted as being scarce, leaving communities with little to no healthcare support for transgender individuals. Formal transgender healthcare training for providers was also seen to be lacking in certifying bodies, with healthcare providers expressing that very little in-class time was spent covering transgender health concerns. One provider expressed this when she reported:

… I don't think that my training was very good in this area (transgender care). I think that there was one course that was an optional course that I took in my master's program and the textbook was “compassionate treatment of gays and lesbians” and nothing else. There was no specific trans awareness in my formal education. … we had a whole diversity course that was supposed to be about cross cultural counselling, and there was a lot of work on the ethnic side and racial side, and the economic things, but no specific stuff around trans-now that I'm thinking about it, it actually didn't exist.

Despite existing barriers, participants in this study viewed Rainbow Health Ontario as having a positive impact on transgender healthcare policy, with the potential to advance policy around gender-affirming surgical care in Ontario. Changes were also seen to be underway with respect to formal transgender healthcare structures, with the opening of the new gender clinic at Sick Kids Hospital in Toronto (opened in 2013). In addition, provider training was noted on individual and organizational levels, in attempts to compensate for the lack of transgender healthcare training on a formal level.

### The meso level

Within a local and interpersonal context, external support (defined as guidance and encouragement provided by others) was found to have a key role in positive healthcare experiences for transgender individuals. Friends, who were often other transgender individuals or allies to the community, were seen as the most important external support, and provided support through giving health-related information and advice, and through sharing experiences. Transgender individuals also reported that friends provided support through acting as mentors, as one participant highlighted,

When I started out I met a couple of friends who became mentors to me. And they were already fairly along in their transition, they were actually living full time as female. And had been for some time and they had all kinds of advice for me. …

All transgender individuals expressed the importance of support groups in their healthcare journeys. Support groups were seen as a place to meet other transgender individuals and build social networks. In addition, many participants indicated that support groups were a place that they could learn about transgender-related resources that existed and share health information about both programs and services that serve the transgender population, and trans-friendly healthcare providers. With respect to the support of family, it was reported that family members did not tend to provide support in relation to providing positive healthcare experiences for transgender individuals. It was noted that family members had varying levels of cisgender bias. One transgender participant expressed this when she stated,

You can't ask your family to provide that support for you because of course, you know, you are working to become something unfamiliar to them, and they don't want you to do that. They want you to remain the way you were, and even if they understand what you're going through, it's difficult.

To provide external support to transgender individuals, mental healthcare providers noted six main ways that they supported their transgender clients to have positive healthcare experiences, which included the following: being a good listener, normalizing the transgender experience, making sure transgender individuals have a good support system in place, helping transgender individuals make informed decisions, writing letters of support when needed, and allowing individuals to take charge of their transition. One provider expressed the need to let transgender individuals take charge of their transition when she indicated:

As a healthcare provider, when you allow somebody to do what they need to do and be who they need to be. … allow them to become who they really want to become and who they are inside, it's an amazing process to watch, and they take care of themselves. You are just enabling a process that they are in charge of, that they are leading, they are dealing with, and as long as they are appropriately resourced economically and socially, and information-wise, they will do it! They will take care of themselves, they will do what they need to do, they will solve their problems. … allowing people to be who they are is so powerful and it almost always works out fine.

In addition, providers felt that going beyond any training they might have received in the healthcare system and self-educating on transgender issues were essential to being a positive support for transgender individuals. For instance, it was noted that being knowledgeable of transgender issues in the media and having a philosophical understanding of identity and gender were considered important factors in providing positive experiences.

Informal networking (defined in this study as the casual conversations that occur among individuals that can result in acquiring useful information in the healthcare setting) was also seen to play a key role in positive transgender healthcare experiences. This networking was seen as a way to learn and share health-related knowledge. Transgender participants reported that they networked with other transgender individuals, using support groups and the Internet as two distinct platforms for building networks.

Friends/family/significant others also used transgender support groups to network and get involved to support transgender individuals they were close with, while healthcare providers used informal networking to establish referral pathways for transgender clients and consult regarding resources and routes of care with other health professionals. One transgender participant reported the importance of informal networking for sharing resources when she stated:

We tend to get together with other girls like us and share information about resources. Somebody will know of a doctor who will prescribe hormones for instance, that person will then become very busy! Because everybody will go there.

Many different pathways through which individuals and organizations networked with one another to share healthcare information were highlighted in this study. Network pathways that were expressed by participants showed the ways in which transgender individuals, the friends/family/significant others they are close with, healthcare providers, transgender and LGBT organizations, and local healthcare organizations were connecting at the meso level, compensating for deficits that exist in larger, macro level structural and policy levels.

In existing network pathways, there was some uncertainty about which local healthcare structures and organizations are networking with one another, and what specific information each individual healthcare organization has. For instance, one friend/family/significant other discussed her concern about whether the local area hospital was aware of the existing services in the area for transgender individuals when she stated:

What kind of resources do they have (at the hospital)? Are they aware of (the local transgender organization)? Are they aware of (the community health center that supports trans people) and (the local AIDS services)? How can we all network so that everybody has the current information and has the knowledge?

### The micro level

On an individual level, it was reported that transgender individuals felt, at times, they were navigating the healthcare system alone. This self-navigation (defined as one finding health system information and services on their own) was not always seen in a positive light; however, it was expressed that self-navigation can lead to more reliable and timely access to health-related information and care. Characteristics for success also emerged as facilitators for successful healthcare experiences. Defined in this study as characteristics expressed by transgender individuals to aid in system navigation, independence, patience, and persistence surfaced as key components in achieving positive healthcare experiences. One transgender participant stressed the importance of patience to have a successful healthcare journey when she stated:

I would basically just sort of get busy with other aspects of my life and put it in the back of my mind and do what I could because I could always go to therapy, I could always go to support groups. And while I was busy doing that and socializing with people I met, then all of the sudden it's time for the next phase. …

Personal strategy development (defined in this study as the development of strategies to achieve and enable positive healthcare experiences) was also reported by both transgender individuals and healthcare providers. Transgender individuals felt that they were often bending the medical establishment to meet their needs, reporting that they were educating their healthcare providers in the hope that these providers would then tend to their needs.

The careful planning of one's healthcare was also used as a strategy by transgender individuals, who recognized that to receive the best care possible, they needed to carefully map their healthcare journey in a way that would best reflect their needs and goals. Through self-managing their healthcare plans, transgender individuals were able to gain a level of control over their healthcare. It was further shown by transgender participants that viewing transgender healthcare as a “do-it-yourself” project was a key factor in having positive healthcare experiences. Given the nature of the current healthcare system, this strategy is important, as one accepts the system for its deficits, and looks internally to compensate for system-level shortcomings through taking initiative and staying informed of changes in the healthcare system.

## Discussion

Healthcare system deficits outlined within the current study are important to discuss because even through the use of a strengths-based approach, one should not discount important deficits that emerge in the research.^28^ Barriers to care that were reported by participants in the current study support existing literature, showing a lack of policy,^2,5,29^ a lack of infrastructure,^2^ and a lack of provider training with respect to transgender healthcare.^5,30^ Despite these barriers, the strengths-based approach allowed the research to go beyond these system barriers and highlight strengths for individuals, friends/family/significant others, and providers who come together in supporting transgender individuals to have positive healthcare experiences.

### The foundation on which positive healthcare experiences are built

Participants in the current study provided an inclusive and holistic view of the ways in which positive healthcare experiences for transgender individuals are facilitated despite macro level barriers.

The mention of provider characteristics when discussing the healthcare experiences of transgender individuals has been referenced in past research, which demonstrates the importance of provider respect^2,5,29,31–33^ and provider knowledge,^2,4,5,22^ by approaching these topics through a deficits-based stance. Transgender participants in the current study did not discuss patient outcomes when asked to define positive transgender healthcare experiences (as prompted through the interview guide), suggesting that a focus on targeting patient experience in combination with health outcomes, rather than outcomes alone, may be of importance.

In reference to previous literature, it has been suggested that patient experience measures relate to health outcomes,^34^ and thus, a positive patient experience may be linked to positive health outcomes and high quality of care. Similarly, in a systematic review, Doyle, Lennox, and Bell found consistent positive associations between patient experience, patient safety, and clinical effectiveness (including outcome measures).^35^ Although further research is needed on the transgender definition of positive healthcare experiences, as well as the association between patient experience and health outcomes for this population, when planning future healthcare initiatives for the transgender population, a focus on targeting patient experience rather than health outcomes might be advisable.

A mental healthcare provider and social worker participated in the current study as a result of being referred by transgender participants. This is significant, indicating the importance that mental health and social work professionals play in creating positive healthcare experiences for transgender individuals. This differs from past literature in this area based in the United States, which has emphasized the role of mental health providers as gatekeepers, with the potential to hinder healthcare access for transgender individuals.^21,36^ It should be stressed, however, that both health professionals in the current study did not function in gatekeeping roles (i.e., were not able to prescribe hormones or make decisions around gender-affirming surgical care). This might have had a significant influence on positive experiences with their transgender clients because these professionals work to support transgender individuals without the requirement to ultimately make decisions around transition-related treatment. In accessing these mental healthcare providers, the socioeconomic status of transgender individuals must also be considered; those without employment benefits or financial means might not be able to access such providers. Implications can be seen for the healthcare system, through which financial support systems for transgender individuals seeking mental health professionals are needed or increased access to mental health support through primary care networks needs to be facilitated.

Both the ways in which transgender participants defined positive healthcare experiences and the ways that providers supported transgender individuals to have positive healthcare experiences lend to the current research in patient-centered care. The patient-centered approach emphasizes the understanding of the patient's world, and reasons for engaging with the healthcare system, in order for the patient and provider to come together in managing the care that the patient receives.^37^ Similar to findings in the current study, the patient-centered approach highlights the connection that the patient and provider have, and allows the patient to be involved in the decision-making process.^38^

Core competencies that have been identified in the patient-centered approach include the following: a welcoming environment, respect for patient's values and expressed needs, patient empowerment, sociocultural competence, coordination and integration of care, comfort and support, access and navigation skills, and community outreach.^39^ Evidently, many aspects of this approach overlap with those identified in building positive healthcare experiences for transgender individuals. For instance, the current study shows that providers who facilitate positive healthcare experiences for transgender individuals create supportive and welcoming environments through displaying respect, and having the ability to establish a connection with clients. Moreover, the awareness of local resources and informal networks that providers in the current study had developed aided them in supporting transgender individuals to effectively navigate the system and access the care they needed.

The value of healthcare providers going beyond any minimal training they may have received in transgender care and having a philosophical understanding of gender and identity shown through this study provide insight into how effective training in social work and mental health professions can be enhanced through the inclusion of gender and identity theory in required curriculum. This supports the work of Benson,^40^ who suggests that gender theory and information pertaining to gender identity are essential in clinical training for therapists. In addition, the finding that providers who support transgender individuals to have positive healthcare experiences are aware of current transgender issues in the media should be taken into consideration in mental health and social work professions. This concept is not entirely new to the literature; sociocultural competence has been outlined as a core competency in patient-centered care,^38^ further emphasizing the important role that the patient-centered approach plays in the positive transgender healthcare experience.

The importance of social support in the lives of transgender individuals has also been shown through previous literature.^41–43^ For instance, higher levels of social support, associated with less anxiety,^41^ and social support from friends and family were among factors that negatively predicted participant's suicidal behavior.^42^ Contrasting existing literature, transgender participants in this study did not report that their family members provided them with much support in navigating the healthcare system. It should therefore be noted that family support, although important to psychological health, was not required in order for transgender participants to effectively navigate the healthcare system to have positive experiences. Participants in the current study, however, did have many other external supports that aided them in achieving positive experiences. One area, less noted in the literature, that needs to be explored further with respect to social support for transgender individuals are the ways through which support is provided to transgender individuals within the context of positive healthcare experiences.

### Networking to effectively navigate through health programs and services

In the context of the healthcare system, and navigation through health programs and services, research looking to the role that networking plays for transgender individuals is limited. Within existing healthcare research from the transgender perspective, networking has been referenced in passing in a limited number of studies. Through the exploration of transgender mental healthcare experiences in the United States, it was found that transgender participants sought referrals from other transgender individuals to find therapists who were knowledgeable and had a good reputation in the transgender community.^40^ Moreover, in a study exploring transgender experiences and interactions with the healthcare system, Sperber et al. found that Internet access is key for networking among transgender individuals and finding information and support.^5^ This finding also lends to the importance of the Internet as a platform for networking among transgender individuals, warranting further research into the role that the Internet plays in healthcare system navigation for transgender individuals.

To date, there has been no research with a specific focus on the informal networking that occurs in relation to transgender healthcare in Canada, nor has there been research that is inclusive in providing more than just the transgender or provider perspectives on networking, allowing for a holistic view of the ways in which networking takes place within the healthcare system. This study provides an introduction of such informal networking; however, a much more in-depth exploration of this area is needed, as the ways in which groups and organizations are effectively connecting with one another to deliver transgender programs and services need to be explored. Through further investigation and network mapping, existing network gaps can be identified and improved in the future.

### Individual strengths and strategies for healthcare success

In the current research, the importance of strengths at an individual level is clear, for it is with these strengths and strategies that transgender individuals were equipped to navigate the healthcare system on a mircolevel. The development of psychological resilience in transgender individuals has been seen as a result of coping with stressful life experiences in relation to gender identity,^44,45^ showing it is not uncommon for transgender individuals to foster internal strengths when presented with barriers. Although there is a lack of research looking to personal strengths of transgender individuals that contribute to positive healthcare experiences, existing deficits-based literature suggests that the particular strengths found in the current study (independence, patience, and perseverance) would be beneficial for transgender individuals, given existing barriers in the healthcare system. Also contributing to resilience in transgender individuals, gender-affirming care such as puberty suppression and hormone treatment has been found to improve psychological functioning in transgender individuals,^46,47^ which likely also plays a role in the perception of positive healthcare experiences. Further research is needed in this area.

The finding that transgender individuals are bending and shaping the Canadian healthcare system to suit their needs is an interesting aspect of the positive transgender healthcare experience. Consistent with existing research, transgender individuals reported that they were educating their own providers; in the current study, however, this was seen as a way to potentially increase the care they would receive. This finding lends to the strengths-based research of Greatheart,^13^ through which the medical establishment can be seen as being “bent” by trans men who found ways to subvert the healthcare system, through means such as the exaggeration of personal experiences to healthcare providers to obtain treatment.

The importance of careful planning of one's healthcare as a result of gaps in current policy, structures, and training in transgender healthcare is a finding that is unique to the current study. This finding provides some insight into the ways that transgender individuals are strategizing to overcome such gaps through planning how they will achieve their healthcare goals in a step-by-step manner. Although there is a current lack of research in this area, existing literature in chronic disease has explored the effects of patient self-management in care. Research shows that health programs that focus on self-management of chronic disease lead to increased self-efficacy and increased health status.^48^ Through emphasizing self-management in chronic disease, the patient becomes the principal caregiver, while the health professional teaches the patient self-management skills, to create a patient-centered system.^49^

For the purposes of this research, the term “active patient participation in care” will be used, because the term self-management can be perceived to coincide with providers teaching patients to manage disease on a clinical level, through medications and treatments. In the current study, however, this concept is being used to describe a wider array of skills, such as system navigation skills and strategies successful communication and collaboration within the healthcare system. Many of the strategies that transgender individuals had developed to navigate the system to have positive healthcare experiences (careful planning of one's healthcare, viewing healthcare as a do-it-yourself project, and self-educating) can be seen as active participation in care. Healthcare providers complement the development of active patient participation in care through helping transgender individuals make informed decisions and letting the transgender individual take charge of their transition. Further research is needed in this area to explore active patient participation in care for transgender individuals in the healthcare setting.

Despite the individual strengths and strategies that are clearly of importance in the positive transgender healthcare experience, the feeling of being alone in the healthcare system must also be addressed. This finding is supported in previous literature.^50^ Although loneliness in the healthcare system should not be interpreted in a positive light, this study shows that, despite feeling alone in the system, transgender individuals are still able to have positive healthcare experiences, which might have significant implications.

## Limitations

The lack of participation by physicians and nurse practitioners in the study is a limitation, as this would have enhanced the current study by providing a medical perspective that was lacking. The current study, however, highlights the important role that mental health professionals play in positive healthcare experiences for the transgender population.

A short time frame was used in the current study to uncover a research area that has remained unexplored. This time frame, however, resulted in a low number of participants, and likely contributed the abovementioned lack of medical professionals in the study. Recommendations for future research in this area involve an extended time frame to include more participants.

Addressing the generalizability of this research, it must be acknowledged that the snowball sampling approach used in this study has been recognized to introduce sampling bias. This sampling approach, however, has been widely used to research “hidden” or hard-to-reach populations,^51^ thus making it appropriate for the current study, which aims to serve as a starting point to uncovering a new area of research to be further explored in the future. Bias that may be evident in the current study as a result of snowball sampling includes a cohort with no transgender individuals of color. This is notable, as past research has shown that transgender individuals of color have increased health risks,^52–54^ which might have implications on how the healthcare system is currently functioning for this population. Furthermore, the snowball sampling approach resulted in all five transgender individuals in this study having an association with a transgender support group, which must be considered, as the importance that transgender individuals in this study placed on support groups may not be generalizable to the wider transgender population. Results of this study many not be generalizable to transgender youth, or the elderly transgender population, which have both been noted to face unique challenges in comparison to the adult transgender population.^55,56^

The use of the strengths-based approach presented challenges within the study. Participants tended to emphasize the negative experiences they had along with the positive experiences (especially on a macro level). The mention of existing deficits by participants when conducting strengths-based research, however, has been noted,^25^ and has been widely shown in transgender healthcare research more specifically. Despite the deficits, this research managed to bring system strengths to the forefront, uncovering system strengths that have previously gone unacknowledged and can be built on in future research and practice.

It is important to note that all data in this study were collected before changes that expanded access for transgender individuals, allowing primary healthcare physicians and nurse practitioners to assess and refer individuals for transition-related funding. It is yet to be seen if this might impact the way in which system-level barriers are perceived by transgender individuals, and whether these recent changes will contribute to positive healthcare experiences for this population.

## Conclusions

Looking to the ways in which transgender individuals in Canada have been supported to have positive healthcare experiences through a strengths-based approach, many new and interesting areas that have previously not been seen in existing literature were uncovered. The supports that facilitate positive healthcare experiences for transgender individuals are seen in meso and micro levels, with emerging changes in some of the macro level systemic barriers that currently exist. Given the small amount of existing literature suggesting that some transgender individuals are satisfied with their healthcare, this study expands upon this research, showing how such a phenomenon takes place in a largely deficits-based system.

## Abbreviations Used

CAMH: Center for Addiction and Mental Health
WPATH: World Professional Association for Transgender Health
